# Wireless Hollow Miniaturized Objects for Electroassisted Chiral Resolution

**DOI:** 10.1021/acs.analchem.3c05544

**Published:** 2024-03-17

**Authors:** Sara Grecchi, Filippo Malacarne, Roberto Cirilli, Massimo Dell’Edera, Sara Ghirardi, Tiziana Benincori, Serena Arnaboldi

**Affiliations:** † Dip. di Chimica, Univ. degli Studi di Milano, Via Golgi 19, 20133 Milano, Italy; ‡ Centro Nazionale per il Controllo e la Valutazione dei Farmaci, Istituto Superiore di Sanità, Viale Regina Elena 299, 00161 Roma, Italy; § Dip. di Scienza e Alta Tecnologia, Univ. degli Studi dell’Insubria, Via Valleggio 11, 22100 Como, Italy

## Abstract

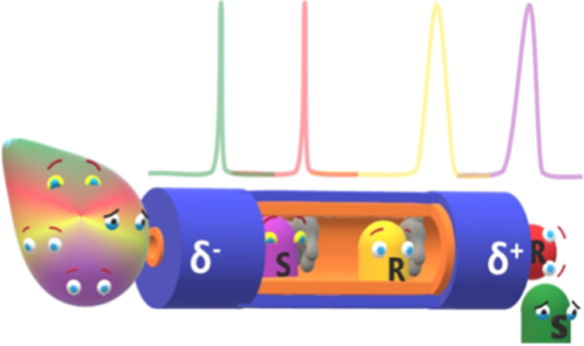

Chiral resolution plays a crucial role in the field of drug development, especially for a better understanding of biochemical processes. In such a context, classic separation methods have been used for decades due to their versatility and easy scale-up. Among the many attempts proposed for enantioselective separation, electroassisted methods are presented as an interesting alternative. Herein, we present the use of wirelessly activated hollow tubular systems for the effective, simple, and tunable separation of racemic and enantioenriched mixtures. These double-layered tubular objects consist of an external polypyrrole chassis, a polymer with good electromechanical properties, functionalized in its inner part with an inherently chiral oligomer. The synergy between the electromechanical pumping process of the outer layer and the enantioselective affinity of the inner part induces the system to behave as a miniaturized chiral column. These hybrid objects are able to separate racemic and enantioenriched solutions of chiral model analytes into the corresponding enantiomers in high enantiomeric purity. Finally, these electromechanical systems can resolve mixtures formed by chiral probes with completely uncorrelated molecular structures injected simultaneously into the single antipodes.

## Introduction

The word chirality originates from the ancient Greek “cheir”, which stands for hands. Chirality is the asymmetry property of an object to exist in two mirror images, called enantiomers. In nature, this feature can be considered as a key aspect, characterizing most materials from nano- to macroscopic length scales. After the discovery of optically active organic compounds able to rotate the plane of polarized light, Pasteur was able to distinguish between the enantiomers of sodium ammonium tartrate salt by isolating the so-called dextrorotary (+) and levorotary (−) isomers.^1^ Commonly, only one of the enantiomeric forms of a chiral molecule can interact with specific enzymes or receptors; thus, each enantiomer can exhibit totally different biological effects. This is one of the reasons why it is mandatory to control the enantiomeric purity of pharmaceutical compounds before commercialization.^2,3^ Modern drug development requires the proper identification, preparation, and characterization of the enantiomers of all bioactive molecules of interest, in order to provide a better understanding of the potential medical application. Even though enantioselective synthesis is an ideal way to overcome separation procedures, in most cases sometimes it is impractical, expensive, and unsustainable.^4−6^ For this reason, generally, enantiomers are obtained from the separation of racemic (equal proportions of the two antipodes) or enantioenriched (asymmetric proportions of the antipodes) mixtures. For decades, gas chromatography (GC), high-performance liquid chromatography (HPLC), and capillary electrophoresis (CE) have been the most widely used separation methods in the pharmaceutical field.^7−10^ In such techniques, the macroscopic output corresponds to a chromatogram or an electropherogram showing differences in retention times or mobilities between enantiomers. The general principle is mainly based on enantioselective interactions between the antipodes of interest and a chiral selector. However, due to the relatively high cost of the equipment and the toxicity of the solvents required, there is strong interest in innovative enantioselective separation methods. In such a context, several unconventional approaches have been developed to overcome the shortcomings described above. For example, microfluidic systems or photoinduced methods have been used for the physical separation of chiral substances.^11,12^ Furthermore, Naaman et al. evaluated the influence of an external magnetic field on the chiral resolution of oligopeptides, oligonucleotides, and amino acids,^13,14^ which is based on the enantiospecific differences in the adsorption rates of the antipodes on the surface of a ferromagnetic material, as a function of the magnetic field orientation.^14^ Alternatively, several efforts have been made to design new enantioselective materials, such as mesostructured heteromembranes,^15−17^ functionalized graphene sheets,^18,19^ or two-dimensional porous metal–organic frameworks,^20−22^ allowing the propagation of only one enantiomer through the pores. More recently, electroassisted enantioselective separation has been achieved by using imprinted metallic surfaces and metallic organic frameworks encoded with chiral information.^23,24^ In both cases, the enantioaffinities of these so-called chiral surfaces are modulated by imposing an electric potential. However, despite the continuous efforts, the development of simple and straightforward methods, competitive with classic chromatographic separation techniques and capable of efficiently resolving racemic, enantioenriched, and complex mixtures (chiral molecules with unrelated composition), is highly desired. An interesting concept to improve enantioselective resolution is exploiting favorable and unfavorable diastereomeric interactions between one of the antipodes of a chiral analyte and an inherently chiral oligomer. Such interactions lead to a differentiation in terms of either thermodynamic redox potentials or crystallization rates, when these chiral π-conjugated macromolecules are used as heterogeneous catalyst or as crystallization template.^25,26^

Recently, the synergy between the electromechanical pumping of polypyrrole, driven by bipolar electrochemistry (BE),^27^ and the enantioselective capabilities of inherently chiral oligomers have been proposed as an interesting alternative for loading, separating, and releasing chiral analytes.^27^ This unconventional hybrid chiral electro-pump, which is wirelessly activated by simply applying an electric field, can tow and release the enantiomers of chiral probes from one extremity to the other, with different retention times. Furthermore, the favorable and unfavorable diastereomeric interactions between the probe and the inherently chiral oligomer allow the separation of racemic mixtures into the corresponding enantiomers with high enantiomeric purity. Herein, we extend this concept by demonstrating the versatility, tunability, and simplicity of these systems in the enantioselective separation of enantioenriched mixtures. In addition, these chiral hollow electropumps have been used for the separation of mixtures containing more than one analyte injected simultaneously as racemates. The results show that the wireless electroassisted chiral tubes can be used as a complementary technique to the classic chromatographic ones in the field of chiral separation for a first screening, where fast and ex situ analyses are required.

## Experimental Section

### Materials

Lithium perchlorate, LiClO_4_ (Aldrich, ≥99.9%), acetonitrile, ACN (Aldrich, ≥99.9% HPLC grade), pyrrole, Py (Aldrich, reagent grade, 98%), sodium dodecylbenzene sulfonate, DBS (Aldrich, technical grade), pH 4 buffer solution (Fluka, prepared with citric acid, NaOH, and NaCl), gold wire (Au, GoodFellow, *d* = 0.3 mm), (*S*)- and (*R*)-carvone (Aldrich, 96 and 98%), (*S*)- and (*R*)-*N*,*N*-dimethyl-1-ferrocenyl-ethylamine (Aldrich, 98 and 97%), solvents for the HPLC (ACN, Aldrich, ≥99.9%, and water, CAS number: 7732-18-5) were used as received. All aqueous solutions were prepared with deionized water (Milli-Q Direct-Q). (*S*)- and (*R*)-2,2′-bis[2-(5,2′-bithienyl)]-3,3′-bithianaphthene enantiomers, named (*S*)- and (*R*)-BT_2_T_4_, were synthesized in our laboratory following a published methodology.^28^

### Synthesis of the Enantioselective Soft Tubes

Wireless tubular devices were designed by following a two-step approach. First, the potentiodynamic electropolymerization of the corresponding enantiopure oligomer (0.75 mM in ACN, 36 cycles, *v* = 200 mV/s) on the surface of a gold wire (Au, *d* = 0.3 mm) was carried out in a classic three-electrode electrochemical cell, using a Pt wire and an Ag/AgCl as counter and reference electrodes, respectively. After the electrooligomerization, the gold wire covered by the enantiopure inherently chiral film was washed with ACN to remove monomer residuals, dried, and used as support for the polypyrrole electrodeposition. The galvanostatic electropolymerization of pyrrole (0.4 mA for 3600 s) was performed in a 0.2 M monomer, 0.25 M sodium dodecylbenzene sulfonate (DBS) aqueous solution. Afterward, the tube, constituted of polypyrrole + the (*S*)-oligomer or (*R*)-oligomer, was mechanically removed from the gold wire (used as the template) after dipping it in acetone for 15 min.

### Wireless Enantioseparation Experiments of Enantioenriched Mixtures

For the wireless enantioseparation experiments, individual enantiopure soft tubes based on polypyrrole (Ppy), modified at the inner part with an oligo-(*R*)- or oligo-(*S*)-selector, were fixed in the middle of a classic bipolar cell at an inert support. Two graphite feeder electrodes were positioned at the extremities of the cell (5 cm apart) containing a pH 4 buffer +0.2 M LiClO_4_ solution acting as the supporting electrolyte. All of the measurements were carried out at a constant electric field of 1.4 V/cm. The racemate of carvone (50:50) and the corresponding enantioenriched mixtures (*S*/*R* 10:90, 30:70, 70:30, 90:10) were prepared from (*S*)-carvone (CAS number: 2244-16-8) and (*R*)-carvone (CAS number: 6485-40-1) and used as such without further addition of solvents and/or dyes. A drop of a few microliters of each probe solution was approached to the negatively charged side of the inherently chiral soft tube. All of the released fractions were collected from the anodic end using a microcapillary.

Chiral HPLC analyses were carried out with HPLC equipment (Agilent 1260 Infinity II) coupled with a Daicel CHIRALPAK IG-3 column in isocratic reverse phase conditions. The HPLC analyses of carvone racemate and mixtures were performed by injecting 1 μL of each solution in the chiral column, with ACN/H_2_O 50:50 as eluent and 1 mL/min flow. The photodiode array (PDA) detector was operated at a wavelength of 236 nm. The signal intensities have been normalized as described in the Supporting Information.

### Multianalyte Wireless Enantioseparation Experiments

For these experiments, individual enantiopure soft tubes based on polypyrrole, modified at the inner part with an oligo-(*R*)- or oligo-(*S*)-selector were fixed in the middle of a classic bipolar cell at an inert support. Two graphite feeder electrodes were positioned at the extremities of the cell (5 cm apart) containing a pH 4 buffer +0.2 M LiClO_4_ solution, acting as the supporting electrolyte. All of the measurements were carried out at a constant electric field of 1.4 V/cm. The racemates of carvone and *N*,*N*-dimethyl-1-ferrocenyl-ethylamine (50:50, (*S*)-enantiomer CAS number: 31886-57-4 and (*R*)-enantiomer CAS number: 31886-58-5) were mixed and used as such without further addition of solvents and/or dyes. A drop of a few microliters of the solutions was approached to the negatively charged side of the chiral soft tube. All of the released fractions were collected from the anodic end using a microcapillary. The HPLC analysis was carried out by injecting 30 μL of each fraction in the chiral column, with H_2_O/ACN 50:50 as the eluent and 1 mL/min flow. The PDA detector was tuned to a wavelength of 236 nm. The signal intensities have been normalized as described in the Supporting Information.

### Chiral Potentiodynamic Measurements of Carvone and *N*,*N*-Dimethyl-1-ferrocenyl-ethylamine

Carvone (2 mM) and *N*,*N*-dimethyl-1-ferrocenyl-ethylamine (2 mM) were dissolved in a commercial pH 4 buffer solution, with the addition of 100 μL of EtOH in order to increase their solubility. The enantioselectivity tests were carried out by using a GC electrode modified with oligo-(*S*)- or oligo-(*R*)-film. The three-electrode cell consists of GC as working electrode, and Pt wire and Ag/AgCl as counter and reference electrodes, respectively. The modified chiral GC electrodes were prepared potentiodynamically by performing 36 oxidative cycles at 200 mV/s scan rate, starting from the enantiopure monomers of the BT_2_T_4_ molecule (0.75 mM) dissolved in ACN + LiClO_4_ 0.1 M as supporting electrolyte.

## Results and Discussion

**Figure 1:**
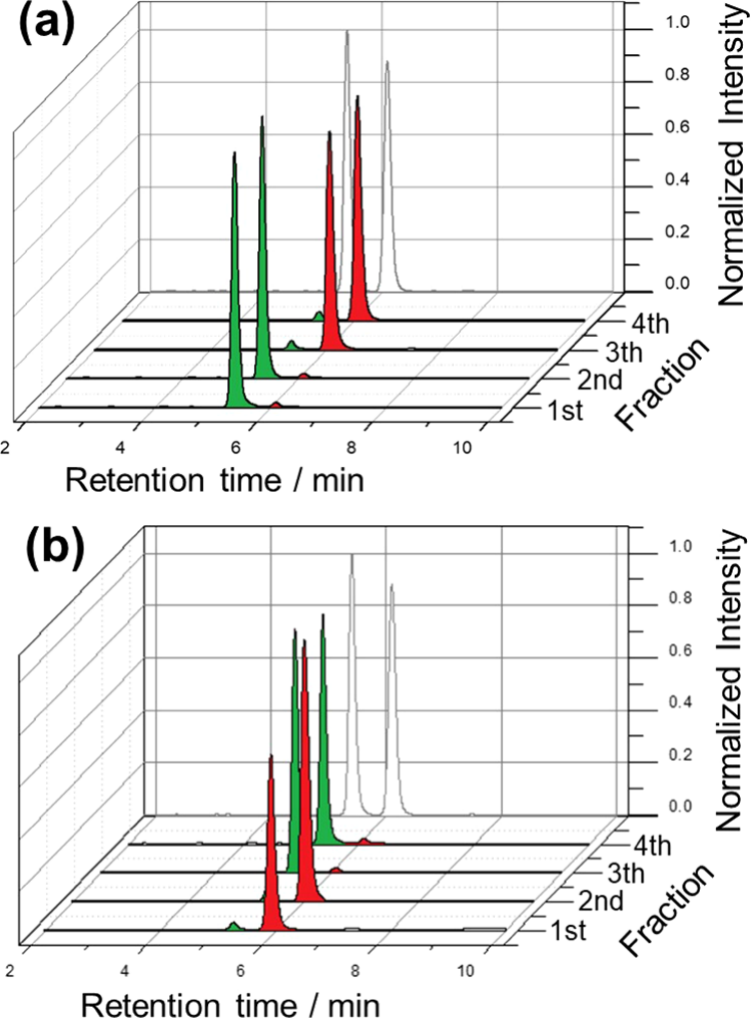
Chromatograms of carvone racemic solutions (50:50) extracted from two independent chiral tubes functionalized at the inner part with the (a) (*R*)-oligomer and (b) the (*S*)-oligomer. For each solution, four fractions were collected with a capillary and analyzed by HPLC. The green and red colors stand for (*S*)- and (*R*)-carvone, respectively. For comparison, the gray lines represent the chromatograms of the racemic carvone prepared and analyzed as such by HPLC.

**Scheme 1:**
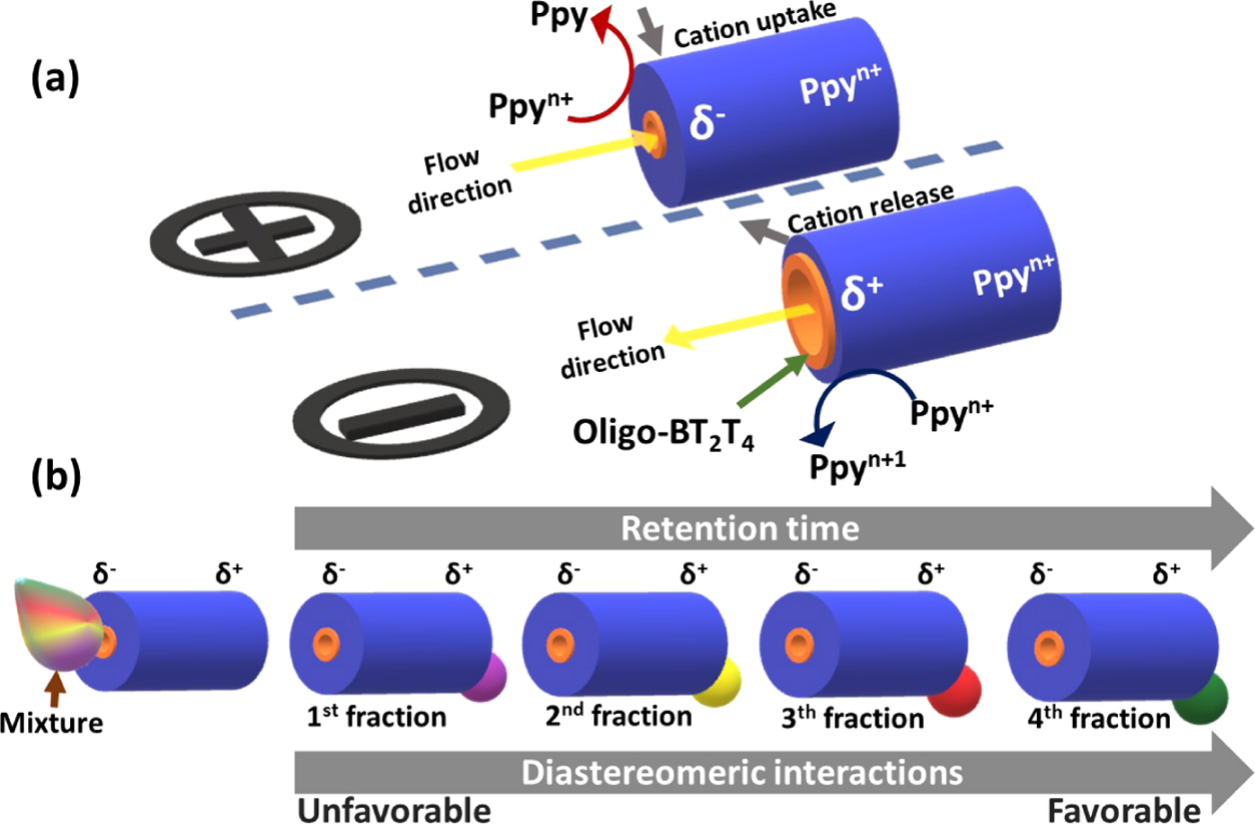
(a) Illustration of the Wireless Electromechanical Mechanism of Fluid Pumping with a Representation of the Cathodic (Top) and Anodic (Bottom) Extremities of a Single Modified Ppy Tube, the Associated Electrochemical Reactions, the Electric Field-Induced Cation Exchange, and the Asymmetric Swelling and Shrinking Process with Its Concomitant Decrease and Increase of Tube Inner Diameter; (b) Illustration of the Electric Field-Assisted Enantioselective Separation of a Mixture of Multiple Chiral Analytes, with a Representation of the Asymmetric Polarization of the Tube and the Corresponding Fractions The orange and blue parts symbolize the Oligo-BT_2_T_4_ and the Ppy film, respectively.

The tubular hollow objects were prepared and functionalized according to the procedure described in a previous work.^29^ The design of the devices consists of a two-step synthetic approach: first, the enantiopure inherently chiral oligomer BT_2_T_4_ was deposited potentiodynamically on the surface of a gold wire used as a template. Subsequently, Ppy (thickness = 39 μm) was galvanostatically polymerized on the BT_2_T_4_ layer. The hybrid tube was then rinsed with deionized water and removed from the metallic template. As stated above, it is possible to induce a wireless electromechanical pumping effect using bipolar electrochemistry (BE). This unconventional technique is based on the asymmetric polarization of a conducting object, or bipolar electrode (BPE), triggered by an external electric field (ε).^30−34^ Under these conditions, a polarization potential difference (Δ*V*) is induced across the BPE generating an anodic (δ^+^) and cathodic (δ^–^) extremity (Scheme 1a). Since in this case, after the electropolymerization, the hollow Ppy tube, acting as BPE, is obtained in its doped state (Ppy^*n*+^), the oxidation and reduction of Ppy^*n*+^ take place at each extremity of the tube, only when the Δ*V* exceeds the thermodynamic threshold polarization potential (Δ*V*_min_) (Scheme 1a).^35^ In general, these redox reactions are associated with the release and uptake of cations, at the anodic and cathodic sides, respectively, to ensure electroneutrality (Scheme 1a).^36−40^ Such a charge-compensating mechanism results in localized swelling and shrinking of the extremities of the tube. Moreover, this wirelessly induced change in volume leads to a decrease and increase in the inner diameter of the cathodic and anodic sides. Thus, in a first-order approximation, such an asymmetric electromechanical phenomenon leads to a unidirectional “pumping effect”, forcing the fluid to pass from the cathodic to the anodic side, in accordance with the Bernoulli principle (Scheme 1a). On the other hand, enantioselectivity is achieved by functionalizing the soft tubes with an inherently chiral oligomer at the inner surface. As mentioned above, due to diastereomeric interactions between these π-conjugated oligomers and the antipodes of a chiral analyte, it is possible to resolve racemates into the corresponding enantiomers. In particular, the inherently chiral oligomer BT_2_T_4_ is well known for its high enantioselectivity in electroanalysis.^25,39,40^ The two α-homotopic sites, located on the two thiophene wings, guarantee the regioregularity of the future structure. Moreover, the high racemization barrier allows the separation of the molecule in the two enantiomers at room temperature. The potentiodynamic synthesis of the enantiopure monomers results in two highly enantiospecific surfaces, named (*R*) and (*S*)-oligo-BT_2_T_4_. As it has been demonstrated previously, the presence of such inherently chiral materials in the hybrid electro-pump permits the enantioselective loading and release of chiral analytes.^29^ Furthermore, such a synergetic effect enables a chiral separation in terms of a difference in retention times. As a matter of fact, a longer retention time of the probe corresponds to favorable interactions between the chiral material (or selector) and one of the antipodes of the chiral analyte, whereas an immediate release is associated with unfavorable interactions (Scheme 1b). Thus, according to Scheme 1b, the analytical probe is swallowed up at the δ^–^ tube end and released at the δ^+^ extremity due to the electromechanical pumping effect, while the inner surface allows the chiral resolution of multiple probes. The enantioselective separation of racemic mixtures of a chiral analyte was carried out to confirm the favorable/unfavorable diastereomeric interactions, as a function of the configuration of the inherently chiral oligomer. Carvone, a monoterpene present in high amounts in caraway, dill, and spearmint essential oil, was chosen as the first chiral model molecule.^41^ In particular, the (*R*)- and (*S*)-antipodes can induce different biological responses, especially toward olfactory receptors. First, conventional electrochemistry was used to test the enantioselectivity of the chiral selector with carvone antipodes, in order to identify the match/mismatch oligomer/carvone enantiomer configuration. Potentiodynamic curves (Figure S1) have shown a peak-to-peak separation between the (*R*)- and (*S*)-carvone of 190 mV with the (*R*)-oligomer-modified electrode. The specular behavior was obtained with the (*S*)-oligomer. After these control experiments, the wireless enantioselective resolution was tested. For this purpose, two independent Ppy tubes, functionalized at the inner surface with the (*R*)- or (*S*)-oligo-BT_2_T_4_, were immobilized on an inert support, at the center of a conventional bipolar cell, as reported in detail in the Experimental Section. The electromechanical pumping effect was achieved by applying a relatively low electric field (1.4 V/cm) in order to trigger only oxidation and reduction of the Ppy^*n*+^ outer layer. This trick avoids possible side reactions in the inner-tube chiral oligomer surface; thus, BT_2_T_4_ is merely responsible for the enantioselectivity. Under these conditions, when a drop of the racemate is placed at the δ^–^ end, the side where the reduction takes place, it is immediately swollen and processed throughout the tube, according to the mechanism described above, and finally ejected from the δ^+^ side. For these experiments, four fractions of the racemic mixture were extracted from the anodic end of the tube by using a capillary. All of the collected samples were filtered and then injected into a high-performance liquid chromatography system (HPLC) equipped with a chiral column, allowing us to prove the composition of each fraction obtained along the full wireless electromechanical pumping. When the racemic carvone solution (50:50) was injected into a tube, functionalized with the (*R*)-oligo-BT_2_T_4_, the first extracted fraction was highly enriched in (*S*)-carvone, in an enantiomeric excess (ee) of 98% (Figure 1a). This result is in good agreement with the unfavorable diastereomeric interaction obtained by classic electrochemistry. Furthermore, such ee remained constant for the second fraction. After 5 min of continuous electrically assisted pumping, the third and fourth samples were released from the anodic extremity of the tube, which, after HPLC analysis, showed high ee in favor of the (*R*)-carvone enantiomer (ee value 96%) (Figure 1a). The specular results were obtained by injecting the racemate through the tube functionalized with the opposite oligomer configuration ((*S*)-oligo-BT_2_T_4_); thus, the first two fractions were highly enriched with the (*R*)-carvone (ee 96%), whereas the last recovered ones with the (*S*)-probe (ee 98%) (Figure 1b). These results demonstrate the possibility of controlling the elution order of the carvone enantiomers simply by inverting the configuration of the selector covering the inner surface of the tube. However, due to the strong diastereomeric interactions between the π-conjugated oligomer and the proper enantiomer of the chiral probe, it is possible to assume that a fraction of the favored antipode remains inside the chiral hollow electro-pump.

**Figure 2:**
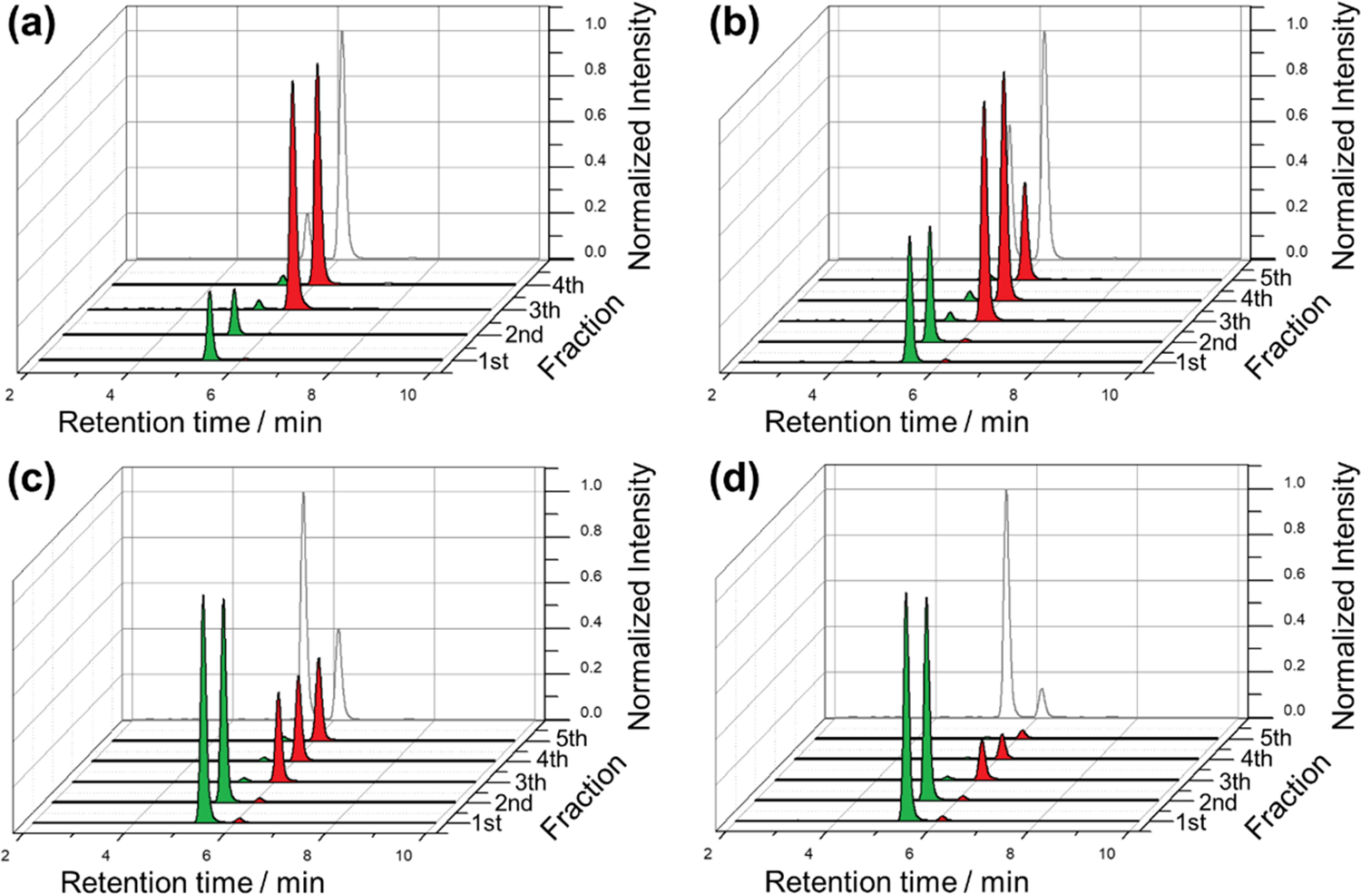
Chromatograms related to carvone enantioenriched mixtures *S*/*R* (a) 10:90, (b) 30:70, (c) 70:30, and (d) 90:10, extracted from the chiral tube functionalized with the (*R*)-oligomer. For each unbalanced mixture, four or five factions were collected by means of a capillary and analyzed by HPLC. The green and red colors stand for the (*S*)- and (*R*)-carvone, respectively. For the sake of comparison, the gray lines represent the chromatograms related to the carvone mixtures prepared and analyzed as such by HPLC.

To evaluate this hypothesis, after each measurement, the tubes were washed with heptane and sonicated in a microvial; the cleaning liquid was then subjected to HPLC analysis (Figure S2). Surprisingly, the chromatogram obtained from the solvent used to clean the tubes shows a barely visible signal related to only one of the enantiomers of carvone, in this case, the (*S*)-one. This test allows concluding that the matching diastereomeric interaction, between the inherently chiral oligomer and its correspondent antipode, inside the tube, only slows down the motion of the right enantiomer, since the unidirectional fluid flow is constant along the entire measurement. Such an effect has been already observed by using imprinted metallic surfaces and metallic organic frameworks encoded with chiral information.^23,24^ After this set of experiments, confirming the wireless enantioselective separation, we have investigated the possible resolution of enantioenriched mixtures, artificially prepared in four different ratios: *S*/*R* 10:90, 30:70, 70:30, and 90:10 and analyzed by HPLC, equipped with a chiral column (Figure S3a). The histograms in Figure S3b were obtained by evaluating the areas related to the chromatograms in Figure S3a. It is visible that the calculated ratios are in good agreement with the theoretical values. In this light, the four enantioenriched solutions of carvone were analyzed and treated separately with tubes functionalized with either (*S*)- or (*R*)-oligo-BT_2_T_4_. Again, the two independent BPEs were immobilized on an inert support, according to the procedure described above. Between each analysis, the tube was washed with heptane by sonication, in order to reuse it for the entire set of experiments. Under a constant electric field (1.4 V/cm), four or five fractions of the processed mixture were extracted from the anodic side of the tube by using a capillary. All of the collected samples were filtered and then injected into the HPLC system equipped with a chiral column. The chromatograms obtained after the enantioseparation of the enantioenriched mixtures, extracted from the tube functionalized with the (*R*)-oligomer, are shown in Figure 2. All of these chromatograms demonstrate that the mixtures, passing through the (*R*)-tube, are coherently separated into the carvone enantiomers (Figure 2a–d). As a matter of fact, in the case of the specular carvone ratios (*S*/*R*, 10:90 and 90:10), the first and second fractions are enriched only with the (*S*)-carvone, that is, the one with the shorter retention time (unfavorable diastereomeric interaction). On the contrary, the third and fourth fractions are enriched with the (*R*)-probe, the one with favorable interaction with the (*R*)-selector. For all of the collected samples, the ee remains constant and above 95%, indicating a highly efficient enantioselective separation. Moreover, comparing the chromatograms of the excurrent fractions extracted from the tubes with the pristine initial solutions (Figure 2, gray plot), a perfect match of both ratios was obtained for all of the enantioenriched mixtures. Furthermore, a specular response was observed for the chiral tube functionalized with the (*S*)-oligomer, under the same operative conditions (Figure S4). These results reflect the high sensitivity of this unconventional analytical approach since enantioselective separation was achieved even for relatively low concentrations of one of the antipodes. To further confirm the efficiency and reproducibility of the enantioselection processes, we have calculated the standard deviation values related to the whole data population, i.e., considering all of the enantiomeric excess values obtained in the case of the excurrent fractions with tubes functionalized with *R*- or *S*-oligomer, and used in sequence in multiple experiments. In such a case, the standard deviations resulted to be 97.1 ± 0.5 and 97.2 ± 0.4 for the tube modified with *R*- and *S*-selector, respectively. Considering that the global time of separation was almost the same for all of the measurements, we can assume that the Ppy chassis can be used several times, in repetition tests, because it maintains good electro-mechanic properties at the chosen work potential.

**Figure 3:**
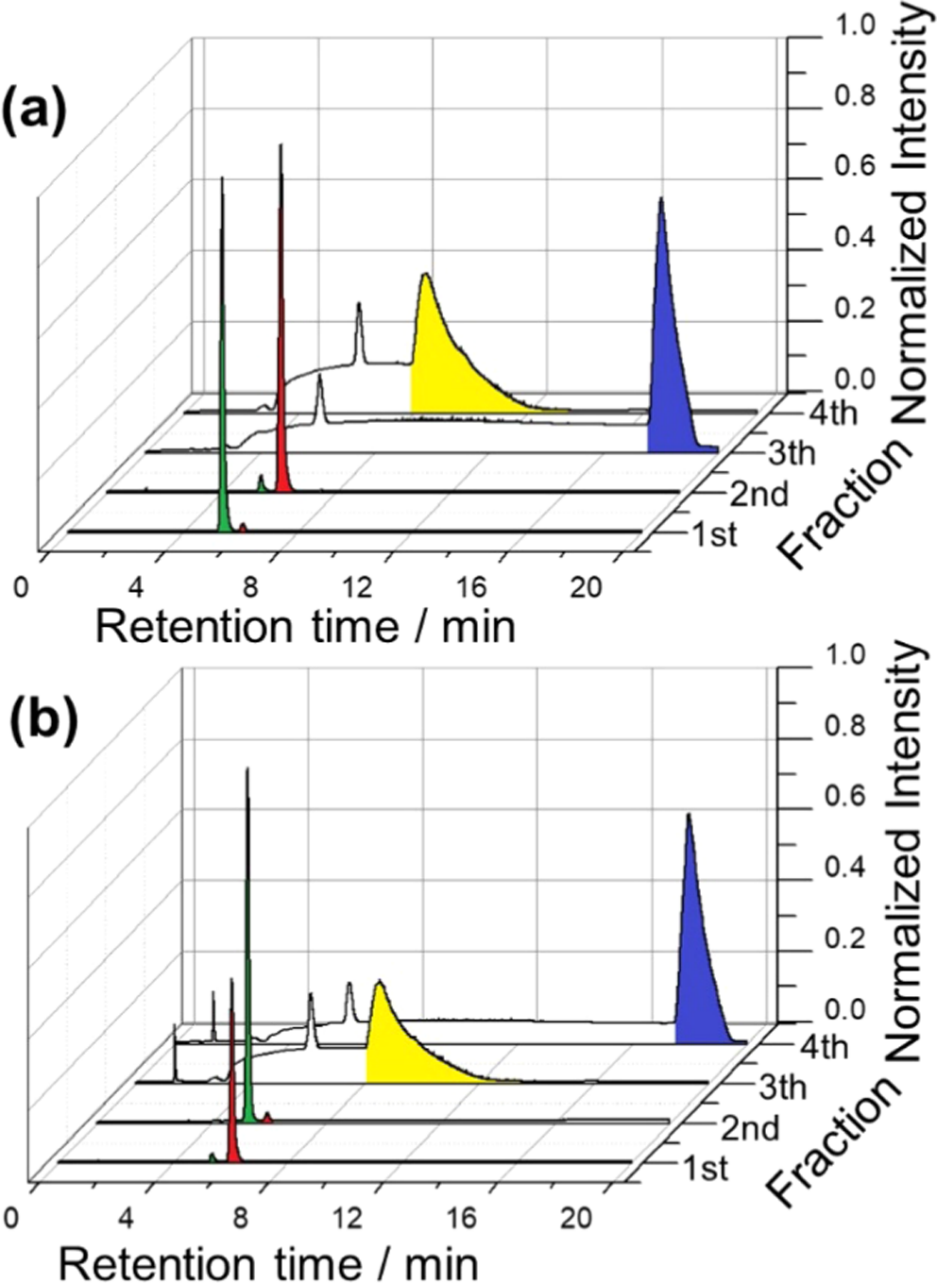
Chromatograms of racemic mixtures of carvone and *N*,*N*-dimethyl-1-ferrocenylethylamine extracted from two chiral tubes functionalized with the (a) (*R*)-oligomer and (b) (*S*)-oligomer. For each solution, four fractions were collected with a capillary and analyzed by HPLC. Green and red colors stand for (*S*)- and (*R*)-carvone, respectively, whereas yellow and blue colors represent (*S*)- and (*R*)-*N*,*N*-dimethyl-1-ferrocenylethylamine, respectively.

Moreover, standard deviations, associated with the enantiomeric excess values, obtained by analyzing the enantioenriched mixtures and related to the second and fourth extracted fractions were calculated to compare the separation efficiency for the *S*- and *R*-tubes. In the case of the object functionalized with the *R*-oligomer, the standard deviations are 98.1 ± 0.002 and 96.7 ± 0.006 for the second and fourth fractions, respectively. In the same manner, for the *S*-tube, the calculated values are 96.6 ± 0.0005 and 97.9 ± 0.0004. Therefore, the tubes are both performing in an efficient way; probably, the *R*-oligomer is less pure than the *S*-one. Finally, we were interested in expanding this concept to the possible enantioselective separation of multiple chiral analytes with different chemical nature and bulkiness. This feature is of great importance since several enantioselective synthetic approaches require organometallic chiral catalysts. Thus, the final reaction mixture usually contains the enantioenriched desired molecule and the antipode of the corresponding catalyst.^42^ In this context, the probes chosen for the resolution tests are carvone and *N*,*N*-dimethyl-1-ferrocenylethylamine (ferrocenyl probe). In particular, the latter is often used in its enantiopure form for asymmetric catalysis.^43^ Moreover, these two molecules are completely different in terms of chemical nature (nonaromatic and aromatic). A mixture of the two analytes, both in their racemic form, was prepared and injected in two tubes functionalized with (*R*)- or (*S*)-oligo-BT_2_T_4_. Wireless electromechanical pumping was carried out, as stated above (Video S1). Once again, the eluted fractions were filtered and analyzed by HPLC. Figure 3 shows the chromatograms related to the analyses of four fractions extracted from soft pumps with opposite configurations of the chiral selector. When the Ppy tube is functionalized with the (*R*)-oligomer, coherently with the previous experiments, the first enantiomer eluted is the (*S*)-carvone (ee 98%), followed by the (*R*)-antipode (ee 96%) (Figure 3a). The third and fourth fractions contain the (*S*)- (ee 94%) and (*R*)-ferrocenyl probe (ee 90%), which are the most retained (the ones with the strongest diastereomeric interactions with the π-conjugated oligomer selector, Figure 3a). The specular chromatograms, with similar ee values for all fractions, were obtained with the tube functionalized with the (*S*)-oligomer (Figure 3b). These results suggest that the chiral oligomer has a higher affinity for aromatic probes since the chiral ferrocene enantiomers are more retained inside of the tube. Furthermore, enantioselective tests, performed with conventional electrochemical experiments, have shown that the oxidation of the *R*-chiral ferrocene on the (*R*)- or (*S*)-oligo-BT_2_T_4_, with matching or mismatching interactions, occurs in a range between +0.4 and +1.4 V vs Ag/AgCl (Figure S5). Thus, by comparing these potential ranges with those corresponding to the oxidation of carvone antipodes (between 1.6 and 2.2 V vs Ag/AgCl), we can conclude that the elution order obtained with the chiral electroassisted pumps is absolutely coherent. Another parallelism can be found by comparing the retention times of the enantiomers of each racemic probe and the differences in terms of peak potential values, obtained from voltammetry experiments. Indeed, in the case of carvone, where the voltammetric separation between the two antipodes is 190 mV, both of the enantiomers are ejected from the chiral tube almost immediately, with a time difference of 5 min. In the case of the ferrocenyl probe, where the voltammetric separation is 1 V, the enantiomers are ejected with a time difference of 15 min.

This general result allows prediction of the elution order of multiple redox chiral analytes by simply using classic electrochemical analysis.

## Conclusions

In conclusion, the aforementioned results demonstrate the possible use of these chiral electromechanical pumps to induce the enantioseparation of multiple chiral molecules of interest. Moreover, it is possible to invert the elution order of the enantiomers in the chiral electromechanical-assisted pumping process since the inherently chiral selector is available in two different configurations. This concept is general and can be used for the enantioseparation of several analytes exhibiting differences in terms of chemical nature (aromatic or nonaromatic) and bulkiness. Such a feature is particularly advantageous when rapid screening is required, since in classical separation techniques, the choice of the proper chiral selector, as well as the optimization of the method, can be time-consuming. In addition, this approach allows enantioenriched mixtures of chiral analytes to be separated into the corresponding enantiomers with high enantiomeric purity, even at relatively low concentrations. Furthermore, efficient enantioseparation of racemic solutions obtained by mixing chiral analytes with completely unrelated molecular structures has been achieved. More importantly, this wireless enantioselective separation system exhibits good reproducibility and reusability since the same soft tube can be used for multiple measurements with high efficiency. Although this is a fundamental proof-of-principle study, the modulation of the fluid flow is possible by controlling the BPE length, diameter, and thickness of both the external and internal layers of the tubes, as well as the electric field. Finally, and most importantly, the proposed concept provides a simple and straightforward way to achieve chiral separations with high enantiomeric purity.

Our device presents multiple advantages in comparison to conventional chiral separation techniques, such as the low cost of the equipment and rapid separation times allowing us to resolve the enantiomers in high enantiomeric purity (ee > 90%). Although this wireless system is not intended to replace common chiral separation techniques, it can be used as a complementary tool for first-time rapid screening.

In our opinion, this work is inspiring for further explorations in the field related to alternative methods for the resolution of chiral analytes.

## Supporting Information Available

Electroassisted pumping process (AVI)
Oligomer selectors in the two configurations were tested toward the enantiomers of carvone; normalized intensities of chromatograms for the first and forth fraction of the collected *S*:*R* 90:10 carvone solution and the chromatogram of heptane solution; chromatograms obtained analyzing five samples of enantioenriched and racemic mixture of carvone enantiomers; oligomer selectors in the two configurations were tested toward the (*R*)-*N*,*N*-dimethyl-1-ferrocenylethylamine (PDF)

## Supplementary Material

ac3c05544_si_001.avi
