# Supplementary material for: Wireless Hollow Miniaturized Objects for Electroassisted Chiral Resolution
Source: Anal Chem. 2024 Mar 17;96(12):4901–8. doi: 10.1021/acs.analchem.3c05544 (PMC10975015; doi:10.1021/acs.analchem.3c05544)
Supplement: Supplementary file 2 — ac3c05544_si_002.pdf [file ac3c05544_si_002.pdf]

Wireless Hollow Miniaturized Objects for Electroassisted Chiral Resolution.

Sara Grecchi,[†] Filippo Malacarne,[†] Roberto Cirilli,[§] Massimo Dell'Edera,[†] Sara Ghirardi,[‡] Tiziana Benincori,[‡] Serena Arnaboldi[†]*

[†]Univ. degli Studi di Milano, Dip. di Chimica, Via Golgi 19, 20133 Milano, Italy.
[§]Centro Nazionale per il Controllo e la Valutazione dei Farmaci, Istituto Superiore di Sanità, Viale Regina Elena 299, 00161 Roma, Italy.
[‡]Univ. degli Studi dell'Insubria, Dip. di Scienza e Alta Tecnologia,Via Valleggio 11, 22100 Como, Italy.

*Corresponding author: serena.arnaboldi@unimi.it

Table of Contents

Enantiomeric excess calculation S2

Additional Data S2

Video of the electroassisted pumping process with the collection of the chiral fractions S4

Enantiomeric excess calculation

The enantiomeric excess (*ee*) of the fractions was calculated by using the following equation:

$$Enantiomeric\ Excess\ (ee)\ = \frac{[(R)E - (S)E]}{[(R)E + (S)E]}\, x\, 100$$

where (*R*)E and (*S*)E are the integrated HPLC peak areas of (*R*)- and (*S*)-enantiomers of the analyte under study.

Additional Data

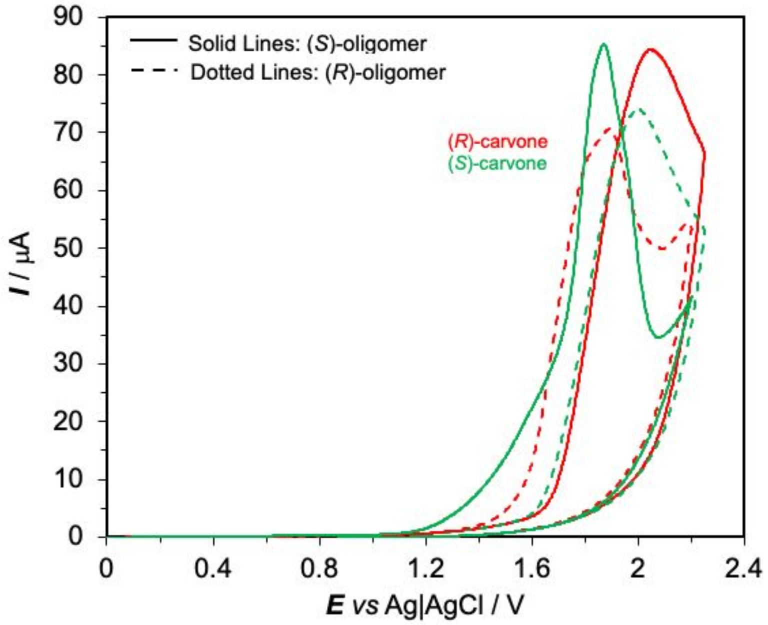

Figure S1. The oligomer selectors in the two configurations ((S) solid line and (*R*) dotted line, respectively) were tested towards the enantiomers of carvone. The red colour refers to the (*R*)-carvone, whereas the green colour to the (*S*)-one.

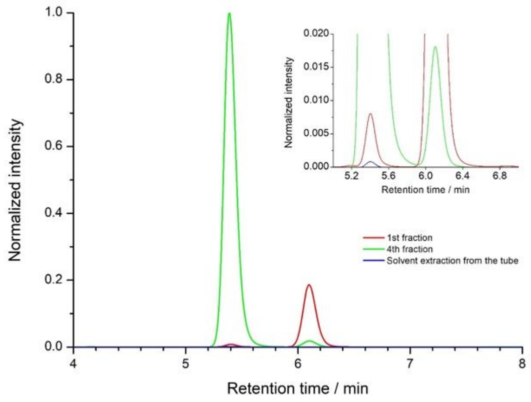

Figure S2. Comparison between the normalized intensities of chromatograms for the first and forth fraction of the collected S:R 90:10 carvone solution, with a tube functionalized with the S-oligomer (red and green line, respectively), and the chromatogram of heptane solution used for washing the tube after the bipolar experiment (blue line in the inset).

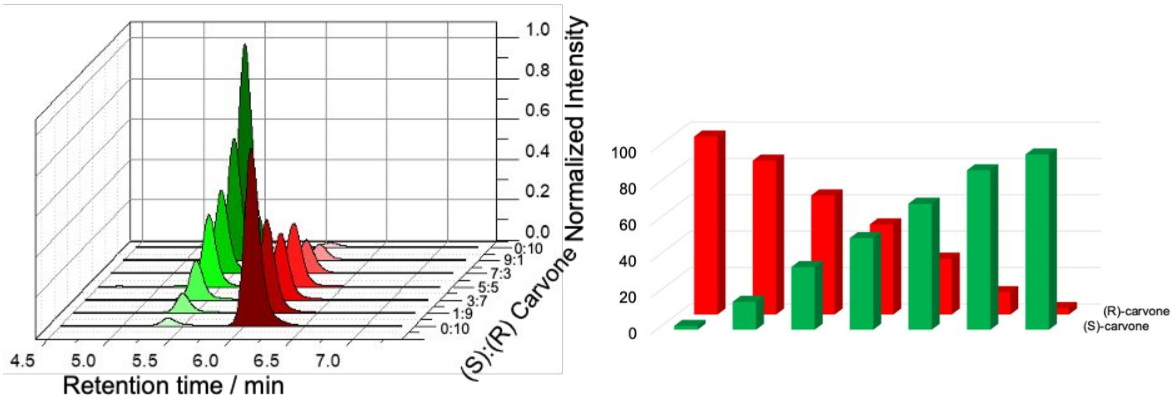

Figure S3. a) Chromatograms obtained analyzing five samples of enantioenriched and racemic mixture of carvone enantiomers in ratios: *S:R* 10:90, 30:70, 50:50, 70:30 and 90:10, for sake of comparison the pristine (*S*)- and (*R*)-carvone enantiomers were injected (dark green and dark red colour, respectively) in the chiral column. b) Histograms related to the analysis of the (*S*)- and (*R*)-carvone peak areas of the chromatograms shown in (a).

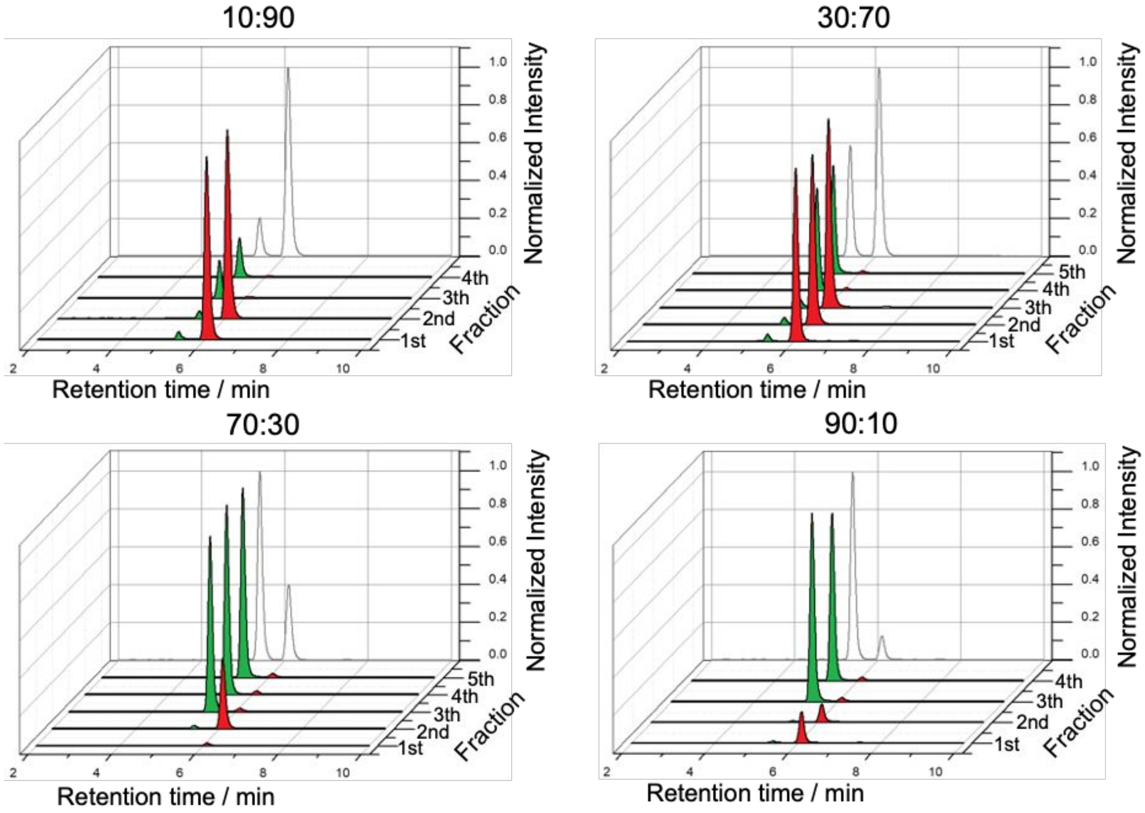

Figure S4. Chromatograms related to the carvone enantioenriched mixtures *S:R* (a) 10:90, (b) 30:70, (c) 70:30 and (d) 90:10, extracted from the chiral tube functionalized with the (*S*)-oligomer. For each unbalanced mixture four or five factions were collected by means of a capillary and analyzed through HPLC. The green and red colors stand for the (*S*)- and (*R*)-carvone, respectively. For sake of comparison, the grey lines represent the chromatograms related to the racemate carvone prepared and analyzed as such through HPLC.

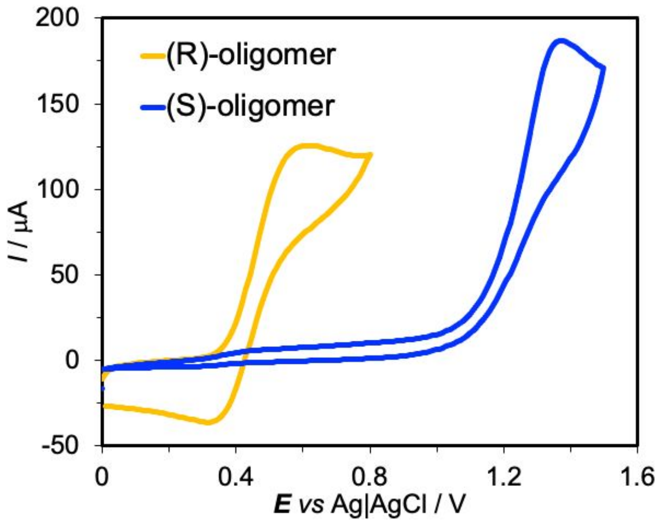

Figure S5. The oligomer selectors in the two configurations ((S) yellow line and (*R*) blue line, respectively) were tested towards the (*R*)- *N,N*-dimethyl-1-ferrocenylethylamine.

Video of the electroassisted pumping process with the collection of the chiral fractions.

Video S1.
